# Spleen-preserving surgery for splenic hydatid cyst: a case report

**DOI:** 10.11604/pamj.2024.47.139.43008

**Published:** 2024-03-26

**Authors:** Tariq Bouhout, Amine Majdoubi, Ramdani Abdelbassir, Badr Serji

**Affiliations:** 1 Surgical Oncology Department, Regional Oncology Center, Mohammed VI University Hospital, Oujda, Morocco,; 2 Mohammed First University Oujda, Faculty of Medicine and Pharmacy Oujda, Oujda, Morocco

**Keywords:** Hydatid cyst, spleen, preserving surgery, case report

## Abstract

Hydatidosis is a zoonosis due to the development of the larval form of Echinococcus granulosus in humans. This disease is very frequent in many countries of North Africa such as Morocco. The most frequent locations of hydatid cysts are the liver (75%) and the lungs (15.4%). Splenic hydatid cyst occurs in only 5.1% of cases. The diagnosis remains challenging and is made upon a hundle of clinical, radiological, biological, and histological arguments. In this paper, we report a case of spleen-preserving surgery for a splenic hydatid cyst to suggest the best management of these hydatid cysts and avoid recurrences.

## Introduction

Hydatid cyst (HC) is a parasitic zoonosis caused by the development of the larval form of *Echinococcus granulosus* [1]. This disease remains endemic in many countries of North Africa such as Morocco [1]. The prevalence of HC is about 1%-7%, with female predominance [2]. HC occurs in the liver (75%) and the lungs (15.4%) and only 5.1% of HC are located in the spleen [3]. In this paper, we report a case of spleen-preserving surgery for a splenic HC to suggest the best management of these HC and avoid recurrences.

## Patient and observation

**Patient information:** we report a 48-year-old male patient, with no medical history and no family history, or tobacco use, who presented with complaints of vague, dull pain in the left upper quadrant.

**Clinical findings:** the physical examination found a left hypochondrial tenderness, without jaundice or signs of hepatobiliary distress, no splenomegaly or lymphadenopathy. No weight loss or change in bowel habits.

**Figure 1:**
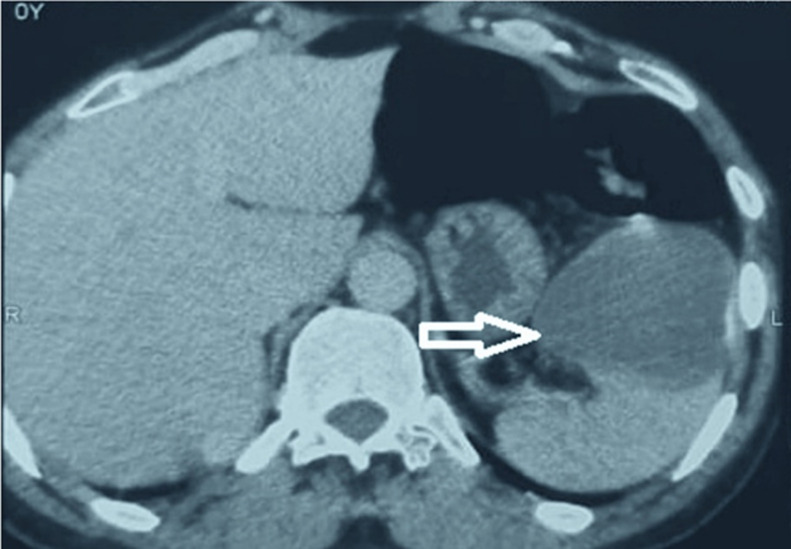
axial abdominal computed tomography (CT) scan showing an uninoculated and well-defined cystic mass in the upper polar of the spleen measuring 115 x 75 mm (white arrow)

**Diagnostic assessment:** abdominal ultrasound showed a splenic cystic mass measuring 11 x 7 cm. Abdominal computed tomography (CT) showed an uniloculated, well-defined cystic mass in the upper polar of the spleen, measuring 115 x 75 mm. The radiological feature is compatible with splenic HC (Figure 1). Hydatid serology was positive.

**Figure 2:**
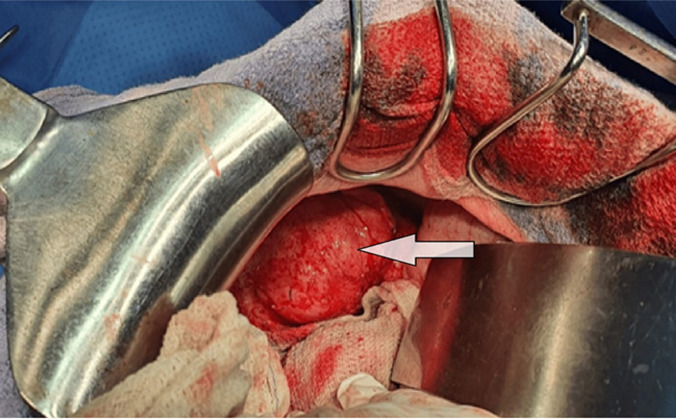
per-operative view of the cystic mass located in the upper pole of the spleen (arrow)

**Figure 3:**
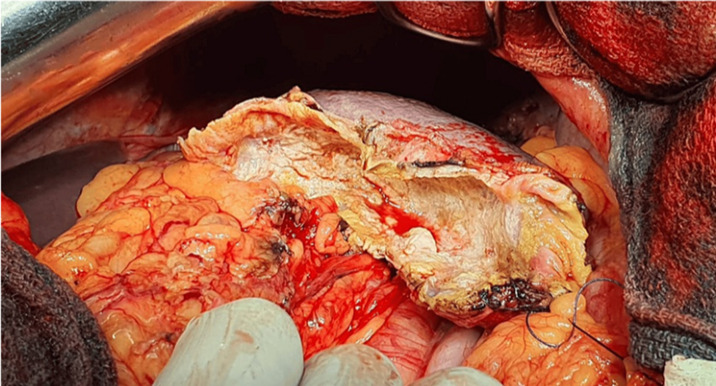
deroofing of the cyst

**Therapeutic intervention:** the patient was admitted to the operating room for surgical exploration. Intraoperatively, we found a voluminous cystic mass in the upper pole of the spleen, and no other cystic lesions were identified anywhere else in the abdomen (Figure 2). The patient underwent deroofing of the cyst with omentoplasty (Figure 3).

The pathological finding was consistent with HC. The post-operative course was uneventful. He was discharged seven days after the operation with a prescription for albendazole (400 mg/day) for three months.

**Follow-up and outcomes:** the thoracoabdominopelvic scan and physical examination demonstrated that there were no recurrences after a follow-up of six months.

**Patient perspective:** the patient declined to give his perspective on her illness.

**Informed consent:** written informed consent was obtained from the patient for publication and any accompanying images.

## Discussion

Hydatid disease is caused by a parasitic infestation by the larvae of one of the *Echinococcal* species, most commonly *Echinococcus granulosus* [4]. Ingesting food or water contaminated with infected dog feces leads to human infection [5].

The clinical presentations of splenic HC are nonspecific and various. Around 30% of cases are asymptomatic and the symptoms depend on the localization, size, and complications due to the development of the cyst [6]. The main symptoms observed are abdominal pain, nausea, loss of weight, and lumbar discomfort [7].

The splenic localization of abdominal HC represents 5.1% [3]. The parasite reaches the splenic parenchyma through the lymphatic system, the bloodstream, or reflux into the spleen from the portal vein during increased intra-abdominal pressure [8]. The rupture of HC in the intra-peritoneal organs or by systemic dissemination may lead to secondary splenic injury [9]. HC of the spleen commonly appears as single or, rarely, multiple anechoic spherical cystic lesions with well-defined limits that can be hyperechoic due to calcifications on abdominal ultrasound [7]. CT scan is more efficient than ultrasound for the detection of HC with the best determination of the number, size, and anatomic location with greater sensitivity (95-100%) [3]. The diagnosis of an HC is based on a range of arguments: clinical, radiological, and serological tests [10].

The treatment options for HC of the spleen include surgery and antihelminthic drugs such as albendazole. Undoubtedly, the ideal treatment is complete surgical excision. Total splenectomy removes the parasitized organ and avoids secondary recurrences. Spleen-preserving surgery helps prevent complications associated with splenectomy especially infections and thromboembolic complications, however, according to many studies, there was no significant difference between total splenectomy and spleen-preserving surgery concerning the recurrence rate [3].

## Conclusion

The diagnosis of splenic HC remains challenging and is made upon a bundle of clinical, radiological, biological, and histological arguments. Spleen-preserving surgery is feasible and represents an alternative to total splenectomy, for the treatment of splenic HC. There is no significant difference between the methods according to studies.
